# Beneficial association of angiotensin-converting enzyme inhibitors and statins on the occurrence of possible Alzheimer’s disease after traumatic brain injury

**DOI:** 10.1186/s13195-020-00589-3

**Published:** 2020-03-27

**Authors:** Mingfei Li, Joel Reisman, Benjamin Morris-Eppolito, Shirley X. Qian, Lewis E. Kazis, Benjamin Wolozin, Lee E. Goldstein, Weiming Xia

**Affiliations:** 1 Center for Healthcare Organization and Implementation Research, Edith Nourse Rogers Memorial Hospital, Bedford, MA USA; 2 Department of Mathematical Sciences, Bentley University, Waltham, MA USA; 3 Geriatric Research Education Clinical Center, Edith Nourse Rogers Memorial Veterans Hospital, Bedford, MA 01730 USA; 4 Department of Health Law, Policy and Management, Boston University School of Public Health, Boston, MA USA; 5 Department of Pharmacology and Experimental Therapeutics, Boston University School of Medicine, Boston, MA USA; 6 Departments of Radiology, Psychiatry, Neurology, and Pathology, Boston University School of Medicine, Boston, MA USA; 7 Departments of Biomedical, Electrical, and Computer Engineering, Boston University College of Engineering & Photonics Center, Boston, MA USA; 8 Boston University Alzheimer’s Disease Center, Boston, MA USA

**Keywords:** Alzheimer’s disease, Traumatic brain injury, Statins, Angiotensin-converting enzyme inhibitors, Treatment, Prevention

## Abstract

**Background:**

Pathological analysis of brain tissue from animals and humans with a history of traumatic brain injury (TBI) suggests that TBI could be one of the risk factors facilitating onset of dementia with possible Alzheimer’s disease (AD), but medications to prevent or delay AD onset are not yet available.

**Methods:**

This study explores four medication classes (angiotensin-converting enzyme inhibitors (ACEI), beta blockers, metformin, and statins) approved by the Food and Drug Administration (FDA) for other indications and evaluates their influence when used in combination on the risk of possible AD development for patients with a history of TBI. We identified patients with history of TBI from an existing Department of Veterans Affairs (VA) national database. Among 1,660,151 veterans who used VA services between the ages of 50 to 89 years old, we analyzed 733,920 patients, including 15,450 patients with a history of TBI and 718,470 non-TBI patients. The TBI patients were followed for up to 18.5 years, with an average of 7.7 ± 4.7 years, and onset of dementia with possible AD was recorded based on International Statistical Classification of Diseases (ICD) 9 or 10 codes. The effect of TBI on possible AD development was evaluated by multivariable logistic regression models adjusted by age, gender, race, and other comorbidities. The association of ACEI, beta blockers, metformin, statins, and combinations of these agents over time from the first occurrence of TBI to possible AD onset was assessed using Cox proportional hazard models adjusted for demographics and comorbidities.

**Results:**

Veterans with at least two TBI occurrences by claims data were 25% (odds ratio (OR) = 1.25, 95% confidence intervals (CI) (1.13, 1.37)) more likely to develop dementia with possible AD, compared to those with no record of TBI. In multivariable logistic regression models (propensity score weighted or adjusted), veterans taking a combination of ACEI and statins had reduced risk in developing possible AD after suffering TBI, and use of this medication class combination was associated with a longer period between TBI occurring and dementia with possible AD onset, compared to patients who took statins alone or did not take any of the four target drugs after TBI.

**Conclusions:**

The combination of ACEI and statins significantly lowered the risk of development of dementia with possible AD in a national cohort of people with a history of TBI, thus supporting a clinical approach to lowering the risk of dementia with possible AD.

## Introduction

Alzheimer’s disease (AD) is the leading cause of dementia and the sixth leading cause of death in the USA. Retrospective cohort studies of the associations between use of selected medications and occurrence of probable AD have been conducted using large claims databases to examine associations between medications that may impact positively with lower risk of dementia and possible AD. These classes of medications include angiotensin-converting enzyme inhibitor (ACEI) [1, 2], simvastatin [3, 4], beta blockers [5], and metformin [6, 7]. Currently, there is no effective treatment to prevent or slow the progression of AD.

Possible association of traumatic brain injury (TBI) with dementia has been extensively investigated. TBI is a broad term defining neurological injury resulting from an external impact involving the head. TBI is a leading cause of long-term disability in the USA and is common in military personnel and veterans, especially in service members with blast exposure from improvised explosive devices. Since 2000, over 350,000 US service members have been clinically diagnosed with TBI; a total of 23% of 4000 soldiers from a brigade combat team deployed in Iraq were reported to have blast-related TBIs [8]. TBI history is associated with significant comorbidities, including posttraumatic stress disorder [9, 10], mood disorders [11], sleep disturbances [12], and increased risk for neurodegenerative diseases such as AD [13] and Parkinson’s disease (PD) [14].

History of TBI has been associated with an increased risk of dementia [15]. A retrospective study of 188,000 veterans found that history of TBI was associated with a 60% increase in the risk of developing dementia [15]. Another retrospective study found a 1.68 times greater risk of dementia in those with TBI after adjusting for sociodemographic characteristics and comorbidities [16]. Several early reports described associations between TBI and AD [17–19]. Lee et al. found a significant association between mild traumatic brain injury (mTBI) and AD based on International Statistical Classification of Diseases (ICD) 9 code in medical records [20]. Based on the data from the National Alzheimer’s Coordinating Center (NACC) that collects AD patients’ data from 39 past and present AD clinical centers (ADC) since 1999 [21, 22], a retrospective study supports a significant association of the early-onset AD with self-reported head injury [23]. A systematic review from 18 studies on the association of TBI severity and AD between 1998 and 2014 revealed that 55% of patients with TBI develop cognitive deficits that meet the clinical diagnostic criteria of AD [24]. At the molecular level, elevated tau-containing neurofibrillary tangle formation and cerebral atrophy are apparent in AD mouse models after TBI exposure compared to mice without TBI [25]. Moreover, progressive spreading of tau proteinopathy has been triggered in wild-type mice by impact or blast TBI [26–29]. Quantitation of tau and phosphorylated tau (p-tau) in mice show that tau and p-tau levels are significantly changed within 1 day after blast, then return to the pre-blast stage when mouse brains were examined at 15 weeks post-blast [30], consistent with previous findings in mini-pigs [31] and mice [32]. Other studies conducted in mice have shown long-term persistence and even progression of blast- and impact-induced p-tauopathy [26–28, 33, 34]. In humans, effort has been made to search for an association (or a lack of) between cognitive decline and overlapping neurodegenerative pathologies [35, 36], and possible biomarkers that reflect those changes [37]. Repetitive mild TBI was found to be associated with tau pathology and allied neurodegenerative changes in the brain [38]. AD-like pathology, amyloid plaques, and neurofibrillary tangles have also been shown to be more prevalent in patients following a single TBI relative to uninjured, age-matched controls [39]. Currently, it is not clear whether TBI-induced molecular changes (such as tau protein phosphorylation) cause irreversible damages leading to AD onset, and equally important, whether available medications can prevent or suppress AD initiation and progression.

On the other hand, analysis of the data from the Religious Orders Study (ROS), Memory and Aging Project (MAP), and Adult Changes in Thought study (ACT) reveals that TBI with loss of consciousness (LOC) is associated with risk for Lewy body accumulation, progression of parkinsonism, and PD, but not dementia, AD, pathologic neuritic plaques, or neurofibrillary tangles [40]. The Adult Changes in Thought data (ACT) is a community-based database to reveal the correlation between clinical characteristics and biochemical/structural features of dementia [41]. Both the Nun study and ACT data were explored to seek correlations between the pathological factors and cognitive function [42]. Similarly, in ROS and the Rush MAP studies, pathology alteration and clinical observations were explored and correlated [43, 44]. A recent study on the dataset from NACC reveals failure of self-reported TBI to predict neuropathologic changes in postmortem brain tissue, indicating that self-reported TBI may not be an independent risk factor for clinical or pathological AD [45]. The self-reported TBI did not predict AD neuropathological changes and was not associated with dementia severity or cognitive function in subjects [45].

In this study, we use an existing VA national database which contains rich longitudinal patient medical information to address two questions, namely, whether TBI contributes to the development of dementia with possible AD, and for those with a history of TBI, whether treatment with ACEI, beta blockers, metformin, statins, or a combination of these medications prolongs the interval between the occurrence of TBI and the onset of possible AD. Previous retrospective cohort studies using claims databases support a protective role for statin treatment with respect to possible AD onset [3]. Therefore, we used statin treatment as the control condition in comparing the association of single and combination of medications on onset of possible AD after TBI. The choices of our medications are based on previously published studies on these medication classes related to AD therapeutics and with the consideration of the sample sizes of our databases. For example, possible benefit of ACEI [2], beta blockers [5], and metformin [7] for AD patients has been explored. Statins with higher blood–brain barrier (BBB) penetration capacity (e.g., lipophilic statins) may have more influence on AD progression [4]. The use of ACEI, beta blockers, metformin, and statins was assessed using Cox proportional hazard models [46] with survival time from initial TBI to occurrence of dementia with possible AD, adjusted for demographics and comorbidities. In previous studies, we found reduced hazard rates or relative risks for dementia occurrence in veterans using ACEI [1] or simvastatin [3]. In this study, we explored the associations of selected concomitant medication use with the occurrence of dementia with possible AD after TBI. We found that combination treatments with ACEI and statins reduce the risk of AD and prolongs the time between the occurrence of TBI and onset of dementia with possible AD.

## Material and methods

### Subjects

This study was approved by the Bedford VA Hospital Institutional Review Board. We used administrative data from the VA Informatics and Computing Infrastructure (VINCI) Resource Center for both inpatient and outpatient visits, vital status, and patients’ prescriptions from October 1, 1998, to April 1, 2018. To extract our study cohort for the analysis of TBI occurrence and AD development, we excluded patients whose age was outside 50 to 89 at the end of the study window. From 1,660,151 VA patients, we applied inclusion and exclusion rules to define our patient and comparison groups for both the TBI model and the medication model. The TBI patient group is comprised of subjects based on ICD-9 and ICD-10 codes (Supplementary Table 1) since October 1, 1998, with two or more outpatient diagnoses or at least one inpatient diagnosis during the study period and at least one medication prescription in the study period. We assembled a control population by random sampling of 10% of VINCI subjects without a claims diagnosis of AD. For subjects taking different medications, the comparison group is the one taking no medication or only statins as a single medication after an occurrence of TBI. TBI patients who initiated any of the four classes of medications before a TBI occurrence and after 1998 were excluded from the study.

### Definition of the survival time (interval between TBI occurrence and AD diagnosis)

In this study, we identified patients with a history of TBI and/or AD based on review of VINCI medical records. Patients who received two or more TBI outpatient diagnoses during the study period (October 1, 1998, to April 1, 2018) were defined as a qualified TBI patient with the first date of diagnosed TBI as the first occurrence date. Based on ICD-9 (331.0) and ICD-10 (G30.x) codes, subjects with Alzheimer’s dementia, AD, primary degenerative dementia of Alzheimer type, or dementia due to AD were included in our study and are collectively termed as those carrying dementia with possible AD. We excluded patients with a diagnosis of dementia with possible AD outside the defined study period or before the occurrence of TBI. Patients with prior recorded diagnosis of AD or with any of the other types of dementia (e.g., vascular dementia, Parkinson’s Disease) were excluded from the study. After a TBI occurrence, a patient who received an outpatient diagnosis of dementia with possible AD or one inpatient diagnosis was considered a TBI patient who developed dementia with possible AD. Our study identified 1057 qualified TBI patients with dementia/AD after a first occurrence of TBI and 13,641 TBI patients without a record of an AD claim diagnosis after an occurrence of TBI during the study period. Survival time was defined as the interval from TBI occurrence to first AD claim diagnosis.

### Medications

Prescription data for ACEI, beta blockers, metformin, and statins were obtained from VINCI. A medication class was included if it was initiated after the first occurrence of TBI and before the diagnosis of dementia with possible AD, or in the control group, before the censoring date. Mortality was included in the model for analysis of survival time. We defined single medication users as patients who initiated at least one of the four target medication classes during the study period. We defined no medication users as patients who did not initiate treatment with medications in any of the four classes after the first TBI diagnosis. The combined medication group was defined as patients who initiated two different drugs after the first TBI occurrence. An initiation of combined medication treatment was considered to be present if the patient was issued prescriptions for two different medications during the study period. Association of angiotensin receptor blockers (ARB) with the risk of dementia has been previously explored [1], and ARB was not included in our analysis as many more subjects use ACEI than those on ARB medications.

### Demographics and covariates

Demographic characteristics (age, race, gender) were included in the study design to adjust for purported effects of these variables and reported comorbidities on TBI occurrence and possible AD diagnosis [47, 48]. Mental health comorbidities included anxiety, bipolar disorder, schizophrenia, posttraumatic stress disorder (PTSD), depression, and substance use disorders. Other medical comorbidities included sleep disorder, thyroid disorder, cardiac dysrhythmia, cancer, congestive heart failure, coronary artery disease, diabetes mellitus, hyperlipidemia, hypertension, liver disease, lung disease, and renal failure. We included the number of TBI events and medication duration as covariates in the model. We defined a new TBI event as one that occurred 60 days after a preceding TBI diagnosis. Medication duration was defined as the total days of prescribed medication.

### Statistical methods

We used logistic regression to assess the effect of first TBI occurrence on possible AD diagnosis [46, 49]. Possible AD diagnosis (ICD-9 331.0 and ICD-10 G30.x) during our study period was the binary outcome in this model, and first TBI occurrence was included in the model as a primary factor for evaluation. Demographics and comorbidities were employed as covariates. Statistical significance was set at an alpha level of *p* < 0.05, and maximum likelihood estimation was used to calculate the odds ratio (OR) with 95% confidence intervals (CI).

We used survival analysis [50] to investigate the association of target medications on the risk of AD diagnosis after the first occurrence of TBI claims diagnosis in the study cohort. Survival time and medication groups were defined as indicated above. Kaplan-Meier curves were used to determine the median survival time from TBI occurrence to diagnosis of dementia with possible AD and to compare cumulative survival between medication groups [51]. Multivariate Cox regression models were used to compare different medication groups while adjusting for demographics and comorbidities [46].

To determine the robustness of the primary association between medication groups and the risk of AD claims diagnosis and to cross-check the consistency of our findings, we conducted several sensitivity analyses. First, we used different reference groups (no medications, statins, or with demographics and comorbidities). Second, we employed a propensity score derived from demographics for adjusting the Cox model, which was entered either as a weighting factor or a covariate [52]. Weigh observations with inverse probability weighting (IPW) is the reciprocal of their estimated probabilities of being observed in event/treatment group [47, 48]. Our study used IPW as a part of a robust check for consistency of results. We also did propensity score as covariates in the regression modeling to test the robustness of our approaches in data analysis. Both are common applications of propensity scores to adjust confounding errors [52]. Third, we performed “leave-one-out cross-validation” (Supplementary Table 2) to cross-check the sensitivity of the primary findings in the medication study.

## Results

### Characterization of study subjects

We have characterized our study cohort and created several groups of subjects. The average age of the dementia patients with possible AD is 80.9 ± 6.7 years, and the average age for the non-AD patients is 70.1 ± 10.1 years. The majority (94.9%) of the study cohort is male. Overall, this study population is composed of 76.2% White, 16.0% African American, 5.4% Hispanic, and 2.4% other ethnic groups (Table 1A).

**Table 1:** Characteristics of subjects

	Possible AD	Non-AD
A. Analysis of association of TBI with development of dementia with possible AD		
Age	80.9 ± 6.7	70.1 ± 10.1
Sex		
Male	62,951	556,525
Female	1388	31,920
Ethnicity		
Caucasian	51,884	445,237
African American	6779	97,782
Hispanic	4565	30,581
Other	1111	14,845
B. Analysis of association of medications with development of dementia with possible AD after TBI occurrence		
Age	76.9 ± 8.5	64.5 ± 9.8
Sex		
Male	310	5663
Female	17	710
Ethnicity		
White	228	4431
African American	35	1243
Hispanic	57	506
Other	7	193
Medication		
No med	92	1957
ACEI	47	644
Beta blocker	35	868
Metformin	29	403
Statin	57	1105
ACEI + beta blocker	15	262
ACEI + metformin	2	133
ACEI + statin	14	359
Beta blocker + statin	21	394
Beta blocker + metformin	3	89
Metformin + statin	12	159

Note: “Medication” designates single and combination patterns of the four target medication classes. Subjects who were prescribed medications other than the target medication classes in the dataset are included as “No med” group. Subjects who were on any of those medication classes at the start of study window or were prescribed three or four of those classes are excluded

We analyzed medication records of our cohort and created the second group of 6700 subjects with medication history of ACEI, beta blockers, metformin, and statins. The average ages for dementia patients with possible AD were 76.9 ± 8.5 years and 64.5 ± 9.8 years for non-AD patients (Table 1B); 89.2% of this population is male (Table 1B).

### Association of TBI incidents with development of dementia with possible AD

**Fig. 1:**
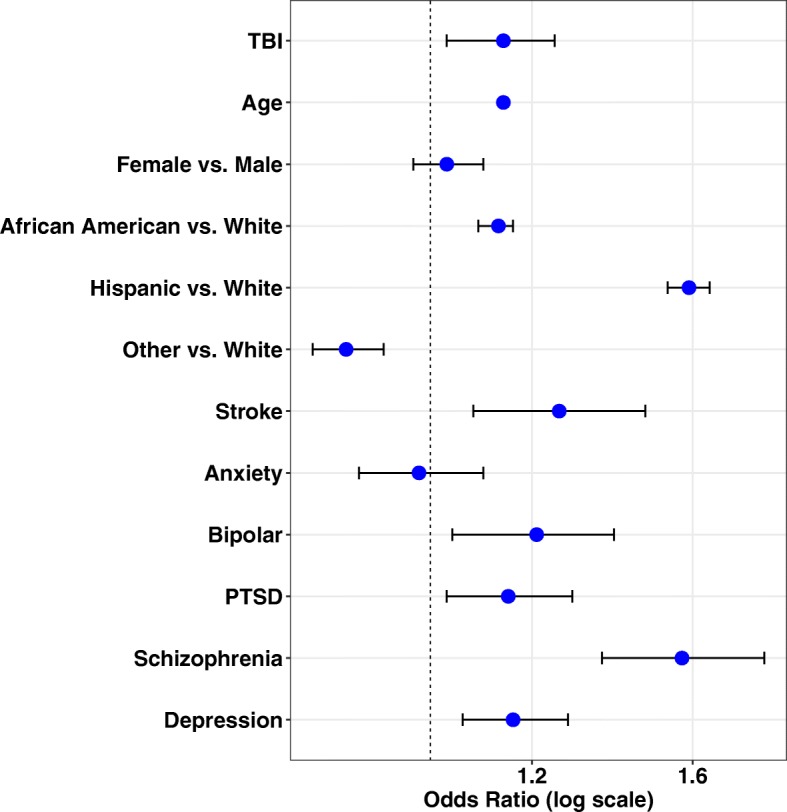
Odds ratio plot for TBI and demographic factors. Odds ratio for developing dementia with possible AD based on demographic factors and history of TBI indicates that prior history of TBI results in a 25% increased chance of developing dementia with possible AD later in life

After controlling for demographics variables and comorbidities, TBI was associated with dementia with possible AD in our study population (Table 2). The odds ratio (OR) of TBI initial occurrence and demographic factors from the logistic model is 1.25 (OR [95% CI] 1.25 [1.134–1.378]) and was statistically significant (*p* < 0.05). Age and ethnicity are the most probable confounding factors and showed positive significant associations with possible AD (*p* < 0.05). Stroke and mental diseases (bipolar disorder, PTSD, schizophrenia, depression) also significantly increased the risk of possible AD (Fig. 1). However, hyperlipidemia, hypertension, alcohol, and drug/substance use disorders showed negative associations likely resulting from collinearity in the models (variance inflation factor > 10). After controlling for the demographic factors and comorbidities, patients with a confirmed TBI initial occurrence was 25% higher in the odds of developing dementia with possible AD, compared with patients who have not experienced TBI (*p* < 0.0001).

**Table 2:** Association of TBI and other factors with the development of dementia with possible AD

	*p* values	Odds ratio	95% confidence interval	
TBI	< .0001	1.250	1.134	1.378
Age	< .0001	1.140	1.139	1.141
Female	0.2739	1.033	0.974	1.096
Black	< .0001	1.130	1.098	1.162
Hispanic	< .0001	1.589	1.534	1.647
Other	< .0001	0.865	0.810	0.923
Sleep	0.3773	0.934	0.803	1.087
Thyroid	0.3708	0.926	0.782	1.096
Cardiac dysrhythmia	0.9049	1.009	0.873	1.166
Cancer (−)	0.0030	0.791	0.677	0.923
Congestive heart failure (−)	< .0001	0.668	0.553	0.808
Coronary artery disease	0.6590	1.027	0.914	1.154
Diabetes	0.3394	1.052	0.948	1.166
Hyperlipidemia (−)	0.0026	0.874	0.801	0.954
Hypertension (−)	< .0001	0.784	0.719	0.856
Kidney (−)	0.0101	0.775	0.638	0.941
Liver	0.2154	0.850	0.657	1.100
Lung (−)	0.0036	0.858	0.774	0.951
Peripheral artery disease	0.3443	1.077	0.923	1.256
Stroke	0.0013	1.291	1.105	1.508
Alcohol (−)	< .0001	0.761	0.671	0.863
Anxiety	0.8961	1.007	0.901	1.126
Bipolar	0.0103	1.208	1.046	1.396
PTSD	0.0058	1.171	1.047	1.310
Schizophrenia	< .0001	1.604	1.387	1.856
Depression	< .0001	1.233	1.123	1.354
Drug substance (−)	< .0001	0.634	0.539	0.746

Note: (−) indicates inverse associations of selected variables

### Influence of ACEI, beta blockers, metformin, statins, and combined therapy on the hazard risk of AD development

From the single medication initiation comparison model, after controlling for demographic variables and comorbidities, we found that beta blockers are negatively associated with the hazard risk of AD development (hazard ratio (HR) [95% CI] 0.36 [0.23–0.57] compared with no medication; 0.56 [0.36–0.88] compared with statin) (Table 3A, B). Patients taking beta blockers are 64% less likely to develop possible AD after an initial occurrence of TBI than patients who are not prescribed these medications (*p* < 0.001, Table 3A). Patients prescribed beta blockers are also 44% less likely to develop possible AD after an initial occurrence of TBI than those taking statins (*p* = 0.01, Table 3B).

**Table 3:** Association of single medication with the development of dementia with possible AD after TBI occurrence

A. Cox regression model (reference group = no medication)								
	*p* values		Hazard ratio		95% confidence interval			
Age	< .0001		1.11		1.10		1.13	
Sex	0.64		1.13		0.67		1.93	
African American	0.28		0.79		0.51		1.22	
Hispanic	< .0001		1.85		1.31		2.61	
Other	0.97		0.99		0.43		2.24	
ACEI	0.01		0.57		0.37		0.87	
Beta blocker	< .0001		0.36		0.23		0.57	
Metformin	0.12		0.68		0.41		1.11	
Statins	0.07		0.70		0.48		1.03	
Medication Duration	0.72		1.00		1.00		1.00	
Sleep disorder	0.58		0.88		0.56		1.39	
Thyroid	0.90		0.97		0.62		1.53	
Cardiac Dysrhythmia	0.07		1.39		0.97		2.00	
Cancer	0.04		0.60		0.37		0.97	
Congestive heart failure	0.03		0.50		0.26		0.94	
Coronary artery disease	0.89		0.98		0.70		1.37	
Diabetes	0.11		1.32		0.94		1.85	
Hyperlipidemia	0.05		1.36		1.00		1.86	
Hypertension	0.99		1.00		0.73		1.37	
Kidney	0.09		0.52		0.24		1.12	
Liver	0.47		1.33		0.61		2.91	
Lung	0.53		0.91		0.67		1.23	
Peripheral Artery Disease	0.35		0.81		0.51		1.27	
Stroke	0.85		0.96		0.65		1.42	
Alcohol	0.93		0.98		0.67		1.44	
Anxiety	0.20		1.24		0.89		1.71	
Bipolar	0.79		1.06		0.68		1.65	
PTSD	0.44		0.87		0.60		1.25	
Schizophrenia	0.80		1.08		0.60		1.94	
Depression	0.01		1.46		1.09		1.97	
Drug substance	0.77		0.93		0.56		1.53	
Number of TBI occurrence	< .0001		1.03		1.01		1.06	
B. Multivariable Cox regression model (reference group = no medication or statin)								
	No med as the reference				Statin as the reference			
Medication	*p* value	HR	95%CI		*p* value	HR	95%CI	
ACEI	0.01	0.57	0.37	0.87	0.44	0.85	0.56	1.29
Beta blocker	< .0001	0.36	0.23	0.57	0.01	0.56	0.36	0.88
Metformin	0.12	0.68	0.41	1.11	0.92	1.02	0.63	1.68
Statins	0.07	0.70	0.48	1.03	–	–	–	–

Note: Models adjusted by demographic variables and comorbidities

**Fig. 2:**
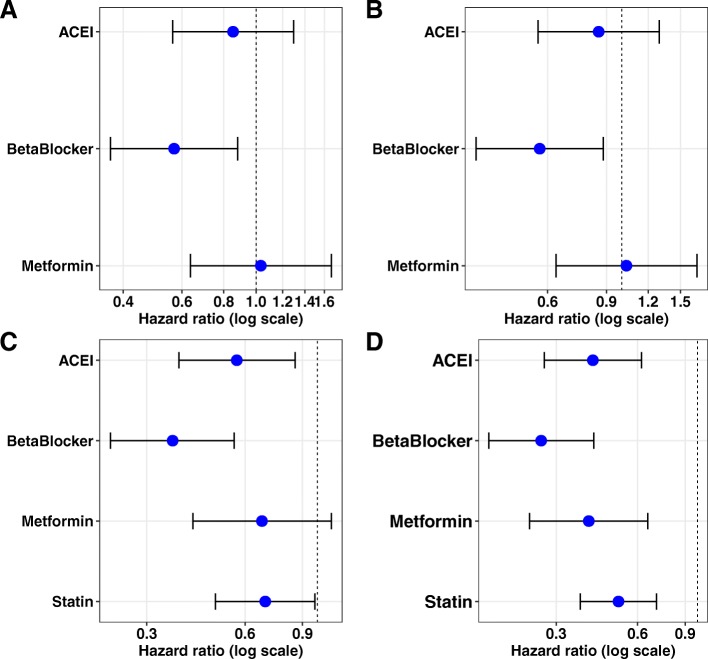
Hazard ratio for association of single medication use with the development of dementia with possible AD after TBI occurrence. **a** Mono medication users were compared to those taking statins using the Cox regression model. Beta blockers show the lowest hazard ratio relative to the statins group. **b** Mono medication users were compared to those taking statins using the Cox regression model with a propensity score adjusted for demographic factors. Beta blockers remain the lowest hazard ratio of developing dementia with possible AD compared to statins. **c** Mono medication users were compared to no medication group using the Cox regression model. **d** Mono medication users were compared with no medication group using the Cox regression model with propensity score adjusted for demographic factors. Beta blocker users have the lowest hazard ratio among all classes of medications analyzed in this study

To check the stability of the results, we included demographic factors and comorbidities in our model and used statin as the reference group. Our results support a significant negative correlation between beta blocker prescription use and AD claims-based diagnosis among single medication users (Fig. 2a). This result is also confirmed by the propensity score-adjusted Cox regression model with demographics and comorbidities (Fig. 2b), where a propensity score of demographic factors is used as an inverse probability weight in the model.

In addition, we included demographic factors and comorbidities in our model and used patients who were not prescribed any target medications as the reference group. Patients who took ACEI or beta blockers as single medication showed a significantly lower hazard risk to develop possible AD after TBI initial occurrence compared to patients not taking any of these medications (Fig. 2c). The propensity score-adjusted Cox regression model (Fig. 2d) confirmed the significance of our outcomes for ACEI or beta blocker treatment.

**Fig. 3:**
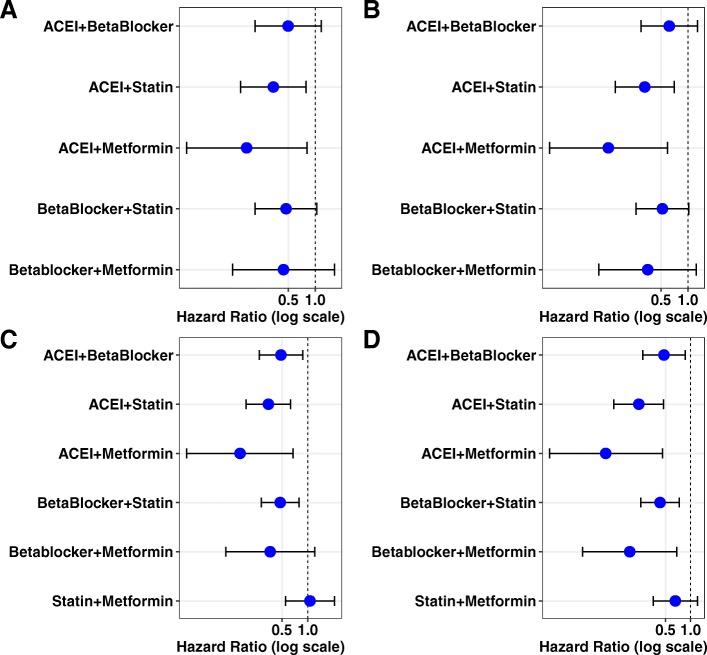
Hazard ratio for association of combined medication use with AD diagnosis after TBI. **a** Patients taking two combined medications were compared to those taking both statins and metformin using the Cox model. The ACEI and statin group showed the most stable reduction in hazard ratio for developing dementia with possible AD. **b** Hazard ratio of same group of patients was analyzed using the Cox model with a propensity score adjusted for demographic factors. **c** Patients taking two combined medications were compared to those taking no medication using the Cox regression model. **d** Hazard ratio from the same group of patients was analyzed using the Cox regression model with a propensity score adjusted for demographic factors

When the multivariable Cox regression model was used to analyze combined medications, ACEI + statins and ACEI + metformin, we found a significantly lower HR compared to the reference group (no medication), with HR [95% CI] 0.44 [0.23, 0.85] and 0.21 [0.05, 0.90], respectively (Table 4A). Since both statin and metformin were previously reported to show a protective, lower risk for the occurrence of dementia [24, 25], the combination of statins + metformin was examined as a reference group. Compared to this reference group, ACEI + statins and ACEI + metformin both exhibited a significantly lower risk, with HR [95%CI] 0.35 [0.15–0.82] and 0.18 [0.04–0.87] respectively (Table 4B). With demographic factors and comorbidities as covariates, results for ACEI + statin combination were consistent (Fig. 3a, c). Models with comorbidities and propensity scores adjusted for demographic factors revealed similar results (Fig. 3b, d).

**Table 4:** Association of combined medications with the development of dementia with possible AD after TBI occurrence

A. Reference group = no medication								
	*p* values		Hazard ratio		95% confidence interval			
Age	< .0001		1.11		1.09		1.13	
Sex	0.42		0.72		0.33		1.58	
African American	0.40		0.78		0.44		1.38	
Hispanic	0.01		1.93		1.19		3.12	
Other	0.68		1.24		0.45		3.38	
ACEI + beta	0.14		0.63		0.34		1.17	
ACEI + statin	0.01		0.44		0.23		0.85	
ACEI + metformin	0.04		0.21		0.05		0.90	
Beta blocker + statin	0.11		0.63		0.35		1.11	
Beta blocker + metformin	0.23		0.47		0.14		1.60	
Statin + metformin	0.45		1.32		0.65		2.70	
Medication duration	0.07		1.00		1.00		1.00	
Sleep	0.55		0.83		0.46		1.52	
Thyroid	0.23		0.62		0.28		1.37	
Cardiac dysrhythmia	< .0001		2.18		1.31		3.63	
Cancer	0.55		0.84		0.49		1.47	
Congestive heart failure	0.38		0.62		0.22		1.78	
Coronary artery disease	0.34		0.76		0.43		1.34	
Diabetes	0.62		0.87		0.51		1.50	
Hyperlipidemia	0.16		1.31		0.90		1.90	
Hypertension	0.09		1.40		0.95		2.06	
Kidney	0.15		0.24		0.03		1.70	
Liver	0.45		0.56		0.13		2.47	
Lung	0.86		1.04		0.69		1.57	
Peripheral artery disease	0.61		1.18		0.62		2.24	
Stroke	0.97		0.99		0.55		1.77	
Alcohol	0.38		1.24		0.77		2.01	
Anxiety	0.32		1.25		0.81		1.94	
Bipolar	0.59		1.20		0.63		2.28	
PTSD	0.11		0.63		0.36		1.11	
Schizophrenia	0.50		1.27		0.63		2.55	
Depression	0.78		1.06		0.72		1.57	
Drug substance	0.07		0.48		0.21		1.08	
Number of TBI occurrence	< .0001		1.04		1.02		1.07	
B. Reference group = no medication or statin + metformin								
	No med as the reference				Statin + metformin as the reference			
Medication	*p* value	HR	95% CI		*p* value	HR	95% CI	
ACEI + beta blocker	0.14	0.63	0.34	1.17	0.16	0.54	0.23	1.27
ACEI + statin	0.01	0.44	0.23	0.85	0.02	0.35	0.15	0.82
ACEI + metformin	0.04	0.21	0.05	0.90	0.03	0.18	0.04	0.87
Beta blocker + statin	0.11	0.63	0.35	1.11	0.09	0.49	0.22	1.11
Beta blocker + metformin	0.23	0.47	0.14	1.60	0.24	0.45	0.12	1.69
Statin + metformin	0.45	1.32	0.65	2.70				

Note: Models adjusted by demographic variables and comorbidities

**Fig. 4:**
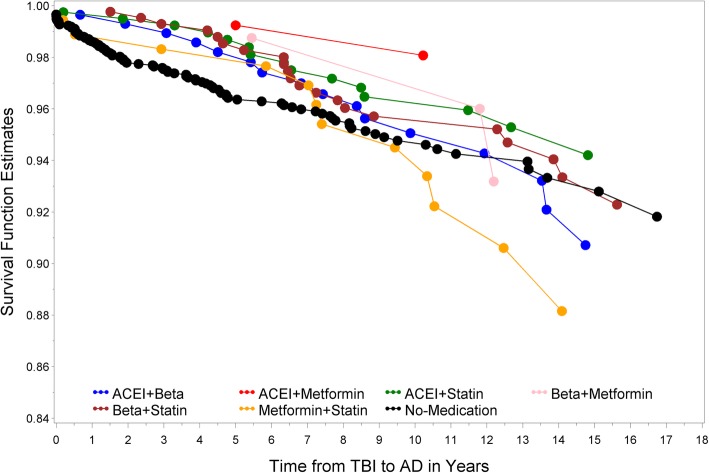
Kaplan-Meier unadjusted survival plot reveals stable and unstable outcomes from different combination of medications. Survival plot was created to illustrate the time from first occurrence of TBI to diagnosis of dementia with possible AD in patients taking no or two combined medications. ACEI and metformin (red) showed the greatest but unstable prolonged time from first occurrence of TBI to diagnosis of dementia with possible AD due to a small sample size. ACEI and statin (green) showed the most stable result for prolonging time from first occurrence of TBI to diagnosis of dementia with possible AD

**Fig. 5:**
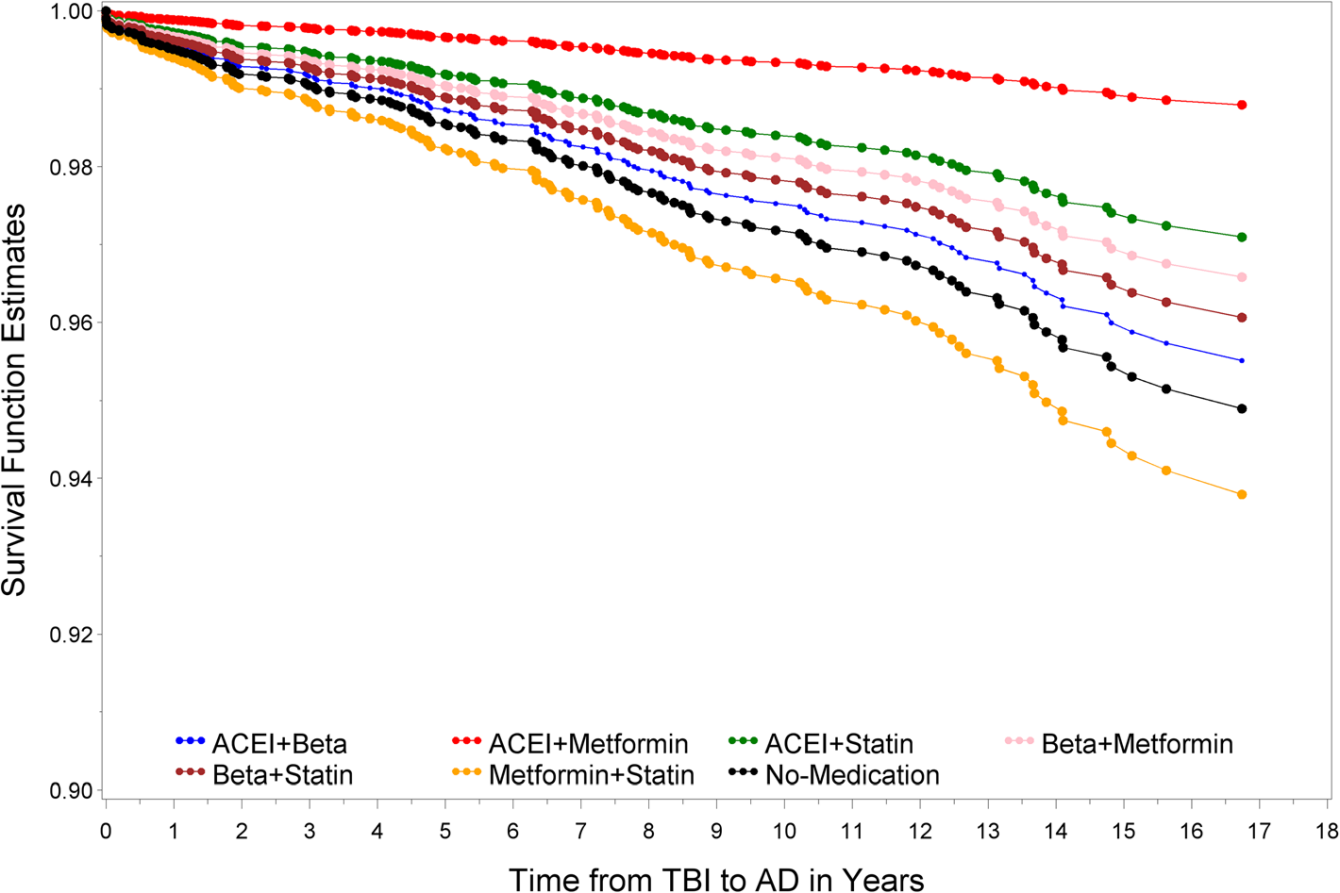
Kaplan-Meier adjusted survival plot reveals rank order of different combinations of medications. Adjusted survival plot was created to illustrate the rank order of two combined medications that affect the time from first occurrence of TBI to diagnosis of dementia with possible AD. Compared to those not taking any medication, patients taking metformin and statin (yellow) appear to develop dementia/AD faster. Except for those taking ACEI and metformin, ACEI and statin users (green) showed the most prolonged time from first occurrence of TBI to diagnosis of dementia with possible AD

The Kaplan-Meier plot for the unadjusted survival time suggested that TBI patients taking ACEI + statins increased the time to claims-based diagnosis of dementia with possible AD (Fig. 4). The adjusted survival time for TBI patients revealed similar outcomes (Fig. 5). Our results suggest that patients taking combined ACEI + statin after the occurrence of TBI lower the hazard risk for possible AD (*p* = 0.01). The number of patients prescribed metformin with other medications is small in our sample population (ACEI + metformin group: *N* = 135, 2% of patients). Consequently, HR confidence interval of this combined medication group is wide (Table 4A and B) and requires a larger sample size for further investigation.

**Fig. 6:**
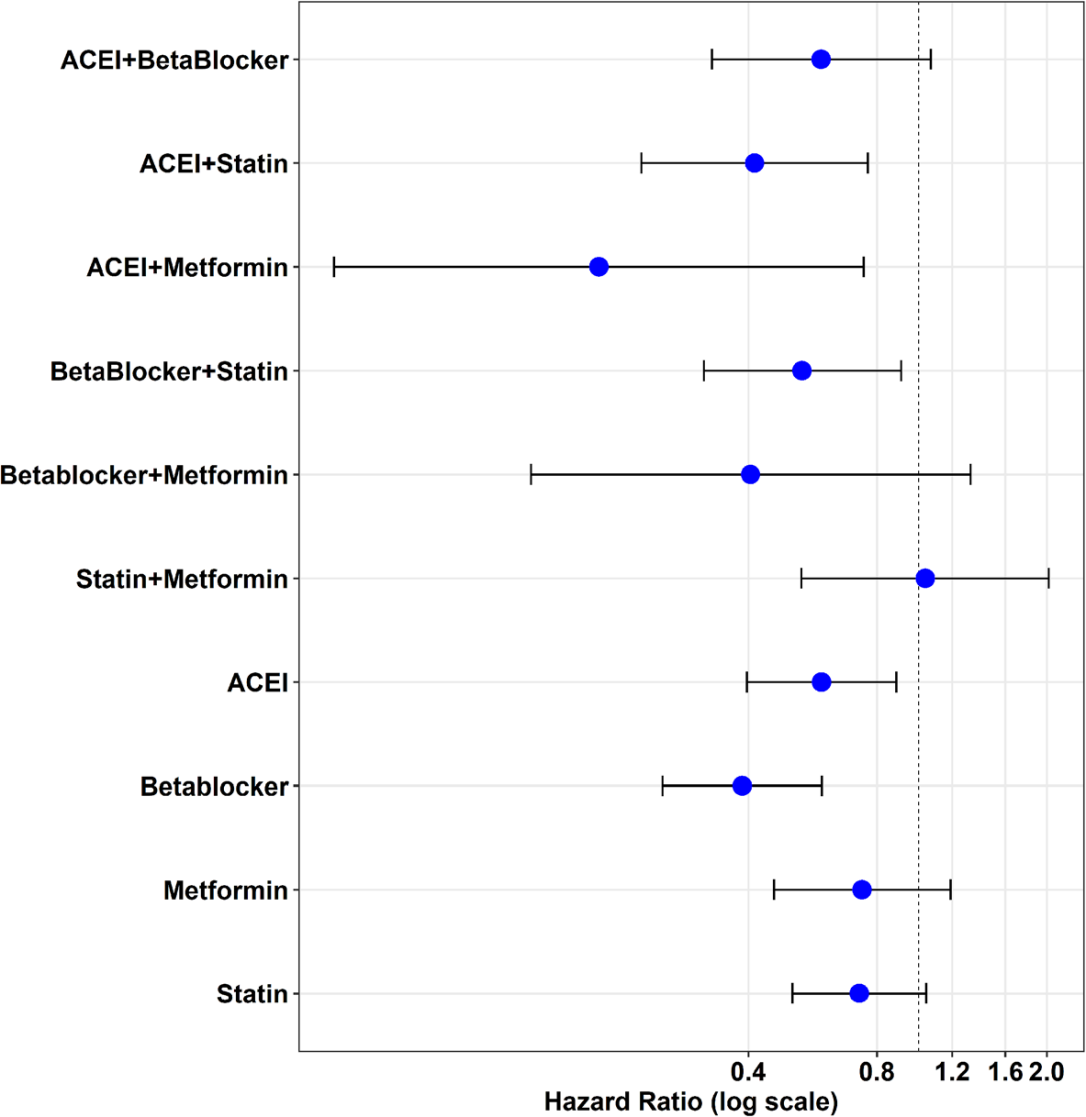
Hazard ratio plot for single and combined medications. Hazard ratio for developing AD was compared between all single and combined medication users and those taking no medication. Due to low sample size, combinations including metformin have a large variation and unstable outcomes. The combination of ACEI and statin reveals the most stable hazard ratio lowering the risk of dementia diagnosis with possible AD after the occurrence of TBI

We also compared both single-agent and combined-agent treatment groups against the no medication group in the adjusted models (Table 5). We found that ACEI and statins as combined medications (*p* = 0.005), ACEI and metformin (*p* = 0.02), and beta blockers (*p* < 0.001) had significantly lower HR for possible occurrence of AD compared to the no medication group (Fig. 6). Given the potential for confounding effect of TBI with that of psychiatric sequelae, we conducted a subcohort analysis to examine effects of combined medication treatment in TBI patients with mental conditions (bipolar disorder, PTSD, or schizophrenia). We found a significant effect of combined treatment with ACEI + statin compared with the no medication group (0.29 [0.19, 0.45], *p* < 0.001).

**Table 5:** Association of single or combined medications with the development of dementia with possible AD after TBI occurrence

Medication	*p* value	Hazard ratio	95%CI	
Age	<.0001	1.11	1.10	1.13
Sex	0.95	0.99	0.59	1.63
Black	0.51	0.88	0.61	1.28
Hispanic	<.0001	1.95	1.44	2.64
Other	0.87	0.94	0.44	2.00
ACEI	0.01	0.59	0.40	0.89
Beta blocker	<.0001	0.39	0.25	0.60
Metformin	0.21	0.74	0.46	1.19
Statins	0.08	0.73	0.51	1.04
ACEI + beta	0.08	0.59	0.33	1.07
ACEI + statin	0.005	0.41	0.23	0.76
ACEI + metformin	0.02	0.18	0.04	0.75
Beta blocker + statin	0.02	0.54	0.32	0.91
Beta blocker + metformin	0.14	0.41	0.12	1.33
Statin + metformin	0.91	1.04	0.53	2.02
Medication duration	0.13	1.00	1.00	1.00
Sleep	0.86	0.97	0.65	1.43
Thyroid	0.60	0.89	0.58	1.37
Cardiac dysrhythmia	0.04	1.42	1.02	1.97
Cancer	0.07	0.69	0.46	1.04
Congestive heart failure	0.03	0.54	0.30	0.95
Coronary artery disease	0.99	1.00	0.74	1.36
Diabetes	0.47	1.12	0.83	1.50
Hyperlipidemia	0.11	1.24	0.96	1.61
Hypertension	0.32	1.15	0.87	1.51
Kidney	0.05	0.47	0.22	1.01
Liver	0.35	1.39	0.70	2.78
Lung	0.47	0.90	0.69	1.19
Peripheral artery disease	0.45	0.86	0.57	1.28
Stroke	0.85	0.97	0.68	1.37
Alcohol	0.86	1.03	0.73	1.45
Anxiety	0.09	1.29	0.96	1.72
Bipolar	0.88	1.03	0.69	1.55
PTSD	0.38	0.86	0.62	1.20
Schizophrenia	0.71	1.10	0.66	1.85
Depression	0.03	1.33	1.02	1.73
Drug substance	0.23	0.75	0.46	1.20
Number of TBI occurrence	0.0002	1.04	1.02	1.06

Note: Duration is the summation of total day supply of single or both medications

To confirm the validity of these findings, we used different statistical approaches to estimate the main effects of the first claims-based occurrence of TBI including multivariable logistic regression models, propensity score-weighted and adjusted multivariable logistic models, and logistic model cross-validation. The major findings from these different approaches were similar, and the significant reduction of the HR by ACEI + statins was consistently found across all methods utilized.

## Discussion

Our study results showed that a history of TBI was associated with increased hazard risk of developing dementia with possible claims-based AD (defined by clinical diagnosis) compared to patients without TBI history. This finding supports emerging data implicating TBI as one of the risk factors for AD [53, 54].

We performed statistical analyses of medical records from a large national VA database of over 1.6 million veterans to investigate the relationship of prior TBI on onset of dementia with possible AD and the therapeutic potential of monotherapy and combination therapy with ACEI, beta blockers, metformin, and statins to postpone onset of dementia with possible AD.

We selected four commonly prescribed medication classes—ACEI, statins, metformin, and beta blockers—based on prior reports suggesting potential prophylactic effectiveness for AD [1, 3, 5, 6]. To estimate the association of two-medication combination therapy on first claims-based diagnosis of dementia with possible AD after initial occurrence of TBI, we used multivariable Cox regression models with propensity score weighting for demographic factors and other covariates. Subjects treated with two-medication combination therapy were compared to reference groups without medication treatment. We performed similar analyses for non-TBI patients. Beta blocker users had significantly lower possible AD risk compared to statin users and to the no medication group in our Cox regression model. Interestingly, the impact of ACEI + statin combination was less clear, and the ACEI + statin combination does not show a significant association with AD risk in the multivariate Cox regression model after controlling for all other variables. Our results suggest that this combined medication regimen is specific for the TBI + dementia/AD population compared to the general population of dementia with possible AD without a history of TBI.

Our analyses indicate that patients prescribed a combination of two of these medication classes were at a lower risk of the initial occurrence of possible claims-based AD after the first claims-based diagnosis of TBI. However, we note one important exception, TBI patients treated with metformin and statins had a claims-based diagnosis of dementia with possible AD sooner than TBI patients in the other medication treatment groups (Fig. 5) and also sooner than TBI patients without medication treatment. This interesting finding may point to an unidentified synergistic pathway that potentiates dementia with possible AD and, as such, may provide clues to mechanisms that link TBI and AD. For individual medication, there was no significant association among metformin users (*p* = 0.12, Table 3B). This result is consistent with a recent study based on pooled analyses from multiple studies [55]. The result for statin was also not significant compared with the reference group (*p* = 0.07, Table 3B). Therefore, there is not sufficient evidence derived from our analysis to support their respective impact on possible claims-based AD after the first claims-based diagnosis of TBI.

Patients taking a combination of ACEI and statins showed prolonged time to development of claims-based diagnosis of dementia with possible AD following TBI. The validity of this finding is supported by other modeling approaches that yielded similar conclusions. Other combinations also had a significant association, namely, the combinations of beta blockers + metformin and ACEI + metformin, albeit exhibited wider variation than ACEI + statins.

There were several important limitations to our study. First, the study identified ACEI + metformin as a combination of possible interest, having the protective, lowest risk ratio in the Cox regression model (HR [95% CI] 0.18 [0.04–0.75]; *p* = 0.02, see Table 5). However, owing to the small sample size of this medication group, this low HR was not estimated with significance as we were limited by the small sample sizes from selected subgroups. Furthermore, we do not have sufficient statistical power for subgroups to adjust for other important covariates including hypertension and diabetes as well as other drugs used for those conditions, since medications often are administered for off-label use and are prescribed for more than one indication in routine practice. A second limitation is that clinical severity measures for TBI are not available in this claims database. In our study, we used two or more clinical visits with diagnosis of TBI as criteria to screen TBI patients for inclusion. This criterion was used as a proxy measure of TBI severity. Currently, it is not possible to create subpopulations among TBI subjects corresponding to hemorrhage, concussion, or other indicators of TBI severity, which is not available in this dataset. Larger datasets with this rich clinical information would be needed in the future. A third limitation is the presence of unmeasured variables in our analysis, including indicators of direct medication adherence and measures related to selection bias due to provider and patient preferences for prescribing specific medications. The significant effect of medication switching (including adding or dropping of one medication) needs to be explored in future investigations. The fourth limitation is that our study was based on a Department of Veterans Affairs database with a population that is predominantly male with a smaller fraction of females, with higher proportion of those veterans with physical and mental health conditions than in a general community-wide population. This would limit the generalizability of the findings. The fifth limitation is the sparse information on biomarkers related to amyloid, tau, and neurodegeneration from the existing database we used. NIA-AA criteria on clinical diagnosis of AD published in 2011 [56, 57] and the guidelines under the research framework published in 2018 refer AD to an aggregate of neuropathologic changes and define it by biomarkers and by postmortem examination in vivo [58]. In this study, we obtained clinical records between 1998 and 2018 with minimum data on amyloid, tau, and neurodegeneration (ATN biomarkers). Information of preclinical AD and genetic information (such as *apoE*) is not available for our analysis, and we could not create a simulation study to predict its influence. Finally, the occurrence of TBI attributable to medications promoting falls was not considered; our analysis only focused on risk of dementia with possible AD after the TBI occurrence.

At the molecular level, our findings of candidate medications (ACEI and statins) to be used in delaying onset of dementia with possible AD are supported by mechanistic studies. Previous studies have shown that ACE activity is elevated in AD and correlates with the Braak stage, and ACE density is 70% higher in the temporal cortex of AD patients relative to control subjects [59]. ACE activity is also correlated with Aβ load in AD patients [60], and cultured neurons exposed to Aβ showed increases in levels and activity of ACE [61]. Population studies have shown that antihypertensive medication usage is associated with reduced occurrence of AD [62]. Statins have been tested as potential therapeutics, as high cholesterol levels are implicated as a risk factor for AD [63]. Cholesterol depletion in neuronal cells resulted in complete inhibition of Aβ formation, and this inhibition was fully reversible upon re-addition of cholesterol to the neurons [64]. Transgenic mice subject to cholesterol-lowering drugs showed reduced brain Aβ peptides and amyloid load by greater than twofold relative to non-treated mice [65]. Additionally, treatment with simvastatin has been shown to reduce Aβ peptide levels in both cellular models and Guinea pigs [66].

In addition to brain pathology manifested in a number of neurocognitive disorders, which are diagnosed using ICD-9 and 10 codes, it is well known that cognitive and brain reserve plays an important role in the maintenance of cognitive function and prevention of neurodegeneration [67]. Difference in cognitive reserve may enable individuals to be more resilient to neural dysfunction, while difference in brain structure may differentiate individuals who may have higher tolerance to brain pathological alteration [67].

Our results have implications in examining FDA-approved drugs for the purposes of testing novel hypotheses and introducing new therapeutics for possible future indications for AD. The challenge of addressing treatment of AD could be targeted in part by the wide-scale use of “big data” sources that have integrated electronic health record information with rich clinical data and provide a valuable data source to link medication use with clinical manifestations. This ultimately gives a viable alternative for future investigations of AD treatment [68]. Similar approaches are already being used in improving detection, treatment, and prevention of complex diseases such as cancer [69] and type 2 diabetes [70]. Further in vivo studies are needed to determine the effectiveness of our drug combinations in Alzheimer’s treatment.

## Conclusion

We have developed a list of FDA-approved drugs with potential effectiveness as therapeutics for dementia with possible AD. This study revealed that the combination of ACEI and statins shows potential in delaying the onset of claims-based diagnosis of dementia with possible AD following the first occurrence of TBI. Future in vitro and animal studies will allow us to establish associations between prescribed medications and effectiveness readouts. Proof of concept studies with those candidate medications will be carried out for consideration in future clinical trials as AD therapeutics. They hold great promise for preventing or delaying disease onset and slowing down its progression.

## Supplementary information

**Additional file 1:Table S1.** ICD-9 and ICD-10 codes used for TBI and AD. Table S2. Leave-one-out cross-validation on Hazard Ratio (reference = No Medication).

## Abbreviations

ACEI: Angiotensin converting enzyme inhibitors
AD: Alzheimer’s disease
Aβ: Amyloid β protein
FDA: Food and Drug Administration
ICD: International Statistical Classification of Disease
IPW: Inverse probability weighting
TBI: Traumatic brain injury
