# Supplementary material for: Beneficial association of angiotensin-converting enzyme inhibitors and statins on the occurrence of possible Alzheimer’s disease after traumatic brain injury
Source: Alzheimers Res Ther. 2020 Mar 27;12:33. doi: 10.1186/s13195-020-00589-3 (PMC7102441; doi:10.1186/s13195-020-00589-3)
Supplement: Supplementary file 1 — Additional file 1:Table S1. ICD-9 and ICD-10 codes used for TBI and AD. Table S2. Leave-one-out cross-validation on Hazard Ratio (reference = No Medication). [file 13195_2020_589_MOESM1_ESM.docx]

Supplementary Tables

Supplementary Table 1. ICD-9 and ICD-10 codes used for TBI and AD

Traumatic Brain Injury

| ICD-9 code |  |
| --- | --- |
| 800.xx | Fracture of vault of skull |
| 801.xx | Fracture of base of skull |
| 803.xx | Other and unqualified skull fractures |
| 804.xx | Fractures involving skull or face with other bones |
| 850.xx | Concussion |
| 851.xx | Cerebral laceration and contusion |
| 852.xx | Subarachnoid subdural and extradural hemorrhage following injury |
| 853.xx | Other and unspecified intracranial hemorrhage following injury |
| 854.xx | Intracranial injury of other and unspecified nature |
| 959.01 | Head injury, unspecified |

| ICD-10 code |  |
| --- | --- |
| S02.0x | Fracture of vault of skull |
| S02.1x | Fracture of base of skull |
| S02.8x | Other specified skull and facial bones |
| S02.91 | Unspecified fracture of skull |
| S06.x | Intracranial injury |

Alzheimer’s disease

| ICD-9 code |  |
| --- | --- |
| 331.0 | Alzheimer’s dementia  Alzheimer’s disease  Dementia of the Alzheimer’s type  Dementia, primary degenerative, Alzheimer type  Primary degenerative dementia of the Alzheimer type |
| ICD-10 code |  |
| G30.x | Alzheimer’s dementia  Alzheimer’s disease  Dementia of the Alzheimer’s type  Dementia, primary degenerative, Alzheimer type  Primary degenerative dementia of the Alzheimer type |

Supplementary Table 2. Leave-one-out cross-validation on Hazard Ratio (reference=No Medication)

| Medication 1 | Medication 2 | Hazard Ratio | |
| --- | --- | --- | --- |
|  |  | 1% percentile | 99% percentile |
| ACEI | beta blocker | 0.515 | 0.527 |
| ACEI | Metformin | 0.167 | 0.171 |
| ACEI | Statin | 0.369 | 0.377 |
| Beta blocker | Metformin | 0.391 | 0.403 |
| Beta blocker | Statin | 0.496 | 0.506 |
| Statin | Metformin | 1.114 | 1.140 |

Note: Models adjusted by demographic variables and comorbidities.
